# Cloning and Spatiotemporal Expression Analysis of *IGF1R* Gene cDNA in *Alopex lagopus* (Arctic Fox)

**DOI:** 10.3390/life15050796

**Published:** 2025-05-17

**Authors:** Wei Xu, Hualin Fu, Xiangyu Meng, Yiwen Sun, Fangyong Ning, Zhiheng Du

**Affiliations:** College of Animal Sciences and Technology, Northeast Agricultural University, Harbin 150030, China; 15937335908@163.com (W.X.); 15069496688@163.com (H.F.); mengxy0706@163.com (X.M.); 15135520115@163.com (Y.S.)

**Keywords:** *Alopex lagopus*, *IGF1R* gene, cloning, sequence analysis, protein characterization, gene expression

## Abstract

This study aimed to clarify the sequence characteristics and spatiotemporal expression patterns of the insulin-like growth factor 1 receptor (*IGF1R*) gene in *Alopex lagopus* (Arctic fox), thereby addressing the existing knowledge gap regarding *IGF1R*-mediated growth regulation in this species. The findings establish a crucial foundation for subsequent investigations into the correlation between this gene and Arctic fox growth traits. Specific primers were designed based on the cDNA sequence of the canine *IGF1R* gene (Accession No. XM_545828). The full-length coding sequence (CDS) of the Arctic fox *IGF1R* gene (1617 bp, encoding 538 amino acids) was successfully cloned via RT-PCR. Phylogenetic analysis using the Unweighted Pair Group Method with Arithmetic Mean (UPGMA) algorithm revealed a 99% sequence homology in the *IGF1R* gene between the Arctic fox and canine, confirmed their closest evolutionary relationship. Protein characterization showed that the *IGF1R* protein has a molecular weight of 60.62 kDa (theoretical isoelectric point pI = 5.15), containing one fibronectin type-III domain and one tyrosine kinase domain, classifying it as an acidic hydrophilic transmembrane protein. Phosphorylation site prediction identified 27 phosphorylation sites, with secondary structures dominated by α-helices (26.39%) and random coils (52.79%). The *IGF1R* gene displayed significant tissue-specific expression variations across 12 examined tissues in Arctic foxes: highest expression levels in testis, minimal expression in stomach, and no detectable expression in duodenum. Spatiotemporal expression analysis revealed that in 2-, 4-, and 6-month-old individuals, hepatic *IGF1R* exhibited a progressive increase, testicular expression reached peak levels at 6 months, and skeletal muscle demonstrated transient upregulation peaking at 4 months. These spatiotemporal expression patterns suggest that *IGF1R* may participate in metabolism and organ developmental processes during critical growth stages of Arctic foxes through tissue-specific regulation.

## 1. Introduction

*Alopex lagopus* is a significant species in the global fur trade, prized for its exceptionally soft texture and pristine fur coloration, which holds irreplaceable value in luxury markets. Body size directly determines pelt surface area—the key grading criterion for commercial valuation—making the breeding of large-sized Arctic foxes a top priority. However, current research on growth regulation remains limited to phenotypic factors, like husbandry conditions (e.g., temperature/humidity) and nutritional management, while molecular genetic mechanisms remain underexplored, hindering the development of precision breeding technologies.

The insulin-like growth factor 1 receptor (*IGF1R*) serves as a central regulator in the vertebrate growth axis, driving cellular proliferation, differentiation, and organ development by mediation in the *IGF-1*/*IGF-2* signaling pathway [1,2]. Functional studies demonstrate that *IGF1R* deficiency results in embryonic developmental retardation [3,4], significantly reduced body size [5], while its overexpression correlates with malignant cellular proliferation [6], collectively establishing its pivotal role in growth-regulatory networks. In mammals, *IGF1R* expression displays distinct tissue specificity and temporal dynamics: during embryogenesis, it shows predominant expression in growth-related organs (liver and skeletal muscle), synchronizes with sexual maturation processes in juvenile reproductive/endocrine organs (testes and pituitary), and transitions to maintaining metabolic homeostasis in adulthood [7,8,9].

Compared to molecular biology research on domestic animals such as pigs and cattle [10], molecular biology research on the Arctic fox remains in its infancy. Current studies are predominantly focused on the regulation of hair follicle development by Wnt signaling pathway genes and the seasonal influence of the *MC1R* gene on coat color [11,12], while research on key growth-axis genes is largely absent. As a canid species, the Arctic fox shares 99% homology in the *IGF1R* gene with canids and exhibits similar growth-regulatory pathways [13]. However, its expression patterns in Arctic fox-specific tissues (e.g., testes and muscle) and developmental stages (e.g., rapid growth phase) remain uncharacterized. Given the *IGF1R* gene’s direct correlation with body size and growth rate in other species, combined with the Arctic fox industry’s urgent demand for larger-sized breeding lines, this gene represents an ideal candidate for elucidating growth regulation mechanisms in this species.

*IGF1R* is a well-established critical gene in mammals; however, whether its expression and functions are conserved in Arctic foxes remains unverified. This study aims to investigate the structural and functional characteristics of the *IGF1R* gene in Arctic foxes, thereby elucidating its role in their growth and development, and to establish a foundation for more in-depth molecular biological studies on this species.

## 2. Materials and Methods

### 2.1. Experimental Materials

#### 2.1.1. Experimental Animals

This experiment was conducted with approval from the Animal Ethics Committee of Northeast Agricultural University. Experimental animals were individually housed in separate cages, fed three times daily with ad libitum access to water. A total of 20 Arctic foxes were used, with 3 individuals sampled at each developmental stage (2-, 4-, and 6-month-old individuals). All animals were provided by the Fur-Bearing Animal Breeding Base of Tuqiang Forestry Bureau in the Greater Khingan Mountains (122°48′ E, 52°48′ N; average elevation 454 m). Euthanasia was performed by electrical shock.

#### 2.1.2. Main Reagents

Molecular cloning system: pMD18-T vector, rTaq DNA polymerase, and restriction endonucleases (Takara Bio, Dalian, China).

RNA extraction system: TRIzol reagent (Invitrogen, Carlsbad, CA, USA), DEPC (Sigma, STL, MO, USA).

Nucleic acid purification system: DH5α competent cells (Tiangen Biotech, Beijing, China), Gel Recovery Kit, and Plasmid Extraction Kit (Tiangen Biotech).

RT-PCR system: PrimeScript RTase and SYBR Premix Ex Taq (Tiangen Biotech).

### 2.2. Experimental Methods

#### 2.2.1. Tissue Sample Collection

Samples were collected at 2, 4, and 6 months of fox growth. At each stage, twelve organ tissues—liver, spleen, kidney, lung, stomach, pancreas, brain, pituitary gland, duodenum, testis, heart, and muscle—were collected for total RNA extraction. All organ tissue samples were immediately flash-frozen in liquid nitrogen and stored at −80 °C until use.

#### 2.2.2. Total RNA Extraction and cDNA Synthesis

Total RNA was extracted using an RNA extraction kit and stored at −80 °C. RNA concentration and purity were measured with a UV spectrophotometer. High-integrity and high-purity RNA was reverse-transcribed into cDNA using a reverse transcription kit and stored at −20 °C.

#### 2.2.3. Cloning, Sequencing, and Bioinformatics Analysis of IGF1R Gene CDS Region

Primers were designed based on homologous sequences from various species (see Table 1). The CDS of the Arctic fox *IGF1R* gene was cloned via PCR, using cDNA derived from liver tissue of 6-month-old individuals. The PCR reaction system (25 µL total volume) included sterile deionized water (16.3 µL), tissue cDNA (2.0 µL), 10× PCR Buffer (2.5 µL), dNTP (2.5 mmol/L, 2.0 µL), forward and reverse primers (10 pmol/µL, 1.0 µL each), and Taq polymerase (0.5 U/µL, 0.2 µL). The amplification protocol was as follows: initial denaturation at 94 °C for 5 min; 35 cycles of denaturation at 94 °C for 40 s, annealing at temperatures as listed in Table 1 (80 s, 50 s, and 40 s, respectively), and extension at 72 °C for 60 s; and a final extension at 72 °C for 10 min. PCR products were electrophoresed on 1% agarose gels, purified using a gel recovery kit, ligated into the pMD18-T molecular vector, and transformed. Then, positive clones were selected for sequencing.

Sequencing fragments were assembled using DNAMAN 8.0, and the *IGF1R* gene structure was annotated using the canine reference genome. Cross-species homology alignment was performed via NCBI Nucleotide BLAST. The open reading frame (ORF) was identified using ORF Finder, and amino acid sequences were translated via DNAMAN. Protein conserved domains were analyzed using Protein BLAST 2.2.26; secretory signal peptides were predicted with SignalP3.0. Multiple sequence alignment was conducted using ClustalX1.83, and a molecular phylogenetic tree was constructed via the neighbor-joining method in MEGA 4.0.

Bioinformatics analysis of Arctic fox *IGF1R* included physicochemical properties, hydrophobicity, transmembrane domains, functional domains, phosphorylation sites, secondary/tertiary structures, and protein interactions. Software tools and prediction functions are listed in Table 2.

#### 2.2.4. Spatiotemporal Expression Analysis of IGF1R Gene in the Arctic Fox

qRT-PCR was used to quantify *IGF1R* expression in 12 organ tissues (liver, spleen, kidney, lung, stomach, pancreas, brain, pituitary gland, duodenum, testis, heart, and skeletal muscle) from 2-, 4-, and 6-month-old individuals (*n* = 3). The reaction system (25 µL) included sterile deionized water (16.3 µL), cDNA template (1 µg/µL, 2.0 µL), PCR Buffer (2.5 µL), dNTP (2.5 mM, 2.0 µL), forward/reverse primers (1.0 µL each), and rTaq polymerase (0.2 µL). Amplification conditions were 94 °C for 5 min; 35 cycles of 94 °C for 30 s, 60 °C for 30 s; 72 °C for 40 s; and a final extension at 72 °C for 10 min. Relative expression levels were calculated using the 2^−ΔCt^ method, with *GAPDH* as the reference gene. The analytical method involved calculating *IGF1R* gene expression levels across samples using the 2^−ΔCt^ formula [ΔCt = Ct (IGF1R) − Ct (GAPDH reference gene)]. One-way ANOVA was performed using GraphPad Prism 10.1 to compare mRNA expression levels among samples.

## 3. Results and Analysis

### 3.1. IGF1R Gene CDS Amplification Results

**Figure 1:**
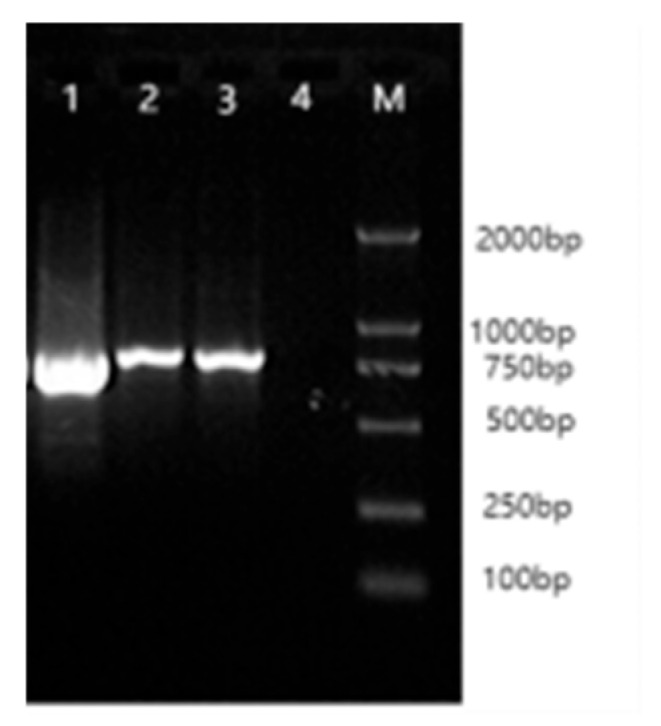
The PCR amplifies results of primers. Lanes 1, 2, and 3: PCR products of primers R*IGF1R*3, R*IGF1R*2, and R*IGF1R*1. Lane 4: control. M: DL 2000 marker.

Total RNA extracted from the organ tissues of Arctic foxes was assessed using Nanodrop spectrophotometry, with A260/A280 ratios ranging between 1.8 and 2.0, confirming that RNA integrity met the requirements for molecular cloning. PCR amplification of cDNA templates using primers R*IGF1R*1-3 generated specific bands of 850 bp, 896 bp, and 727 bp, as verified by 1.5% agarose gel electrophoresis (Figure 1).

### 3.2. Sequencing Results and Sequence Analysis

**Figure 2:**
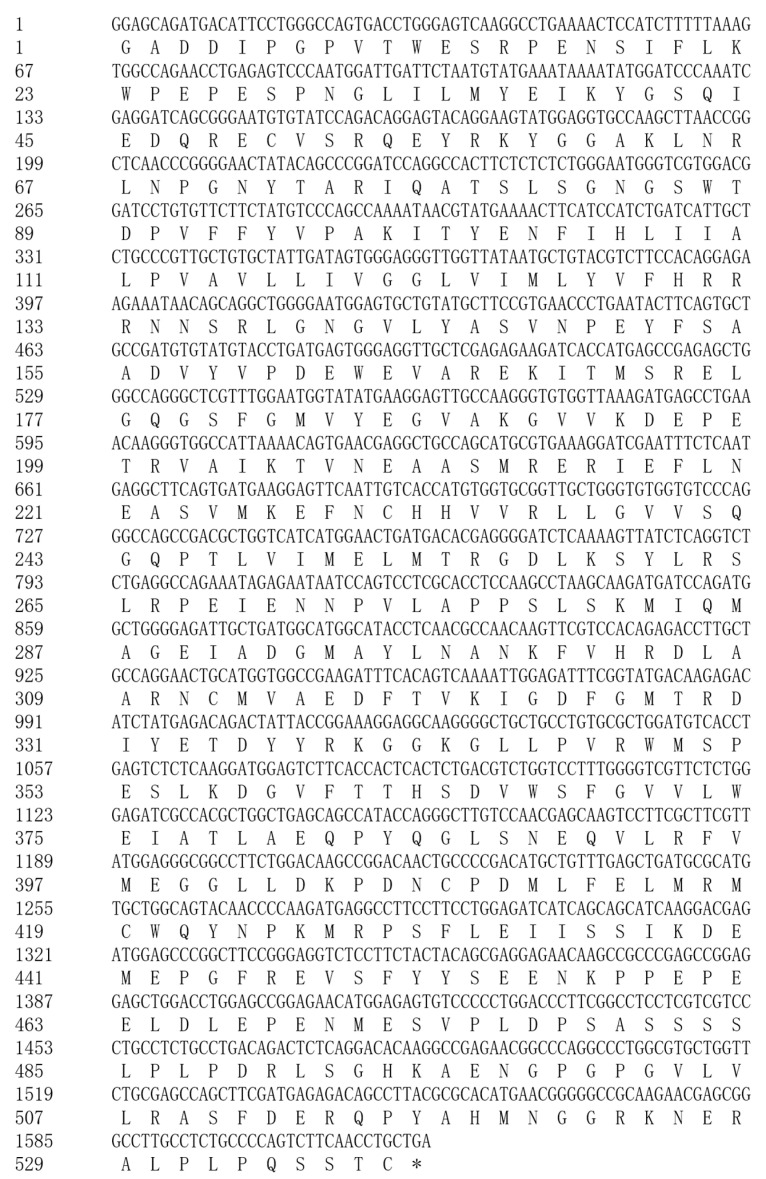
cDNA sequence and putative amino acid sequence of *IGF1R* from the Arctic fox.

Sequencing data assembly yielded a 2411 bp cDNA fragment of the Arctic fox *IGF1R* gene, consistent with the predicted structure of canine *IGF1R*. NCBI BLAST analysis demonstrated cross-species homology: 98% with Canis, 93% with Equus caballus, 91% with Homo sapiens, Macaca mulatta, Sus scrofa domesticus, and Ovis aries, 90% with Ailuropoda melanoleuca and Bos taurus, and 88% with Mus musculus. Phylogenetic analysis confirmed its conservation among mammalian *IGF1R* genes. Partial nucleotide and amino acid sequence alignments are detailed in Figure 2.

### 3.3. Similarity Analysis and Phylogenetic Tree Construction of IGF1R Gene

**Figure 3:**
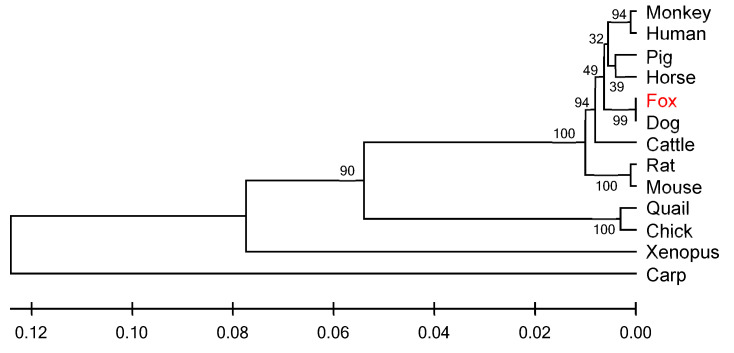
UPGMA phylogenetic tree analysis based on amino acid sequence of *IGF1R* from different species.

Comparative analysis of *IGF1R* amino acid sequences from Arctic foxes and 12 vertebrates (including *Canis*, *Felis catus*, and *Bos taurus*) revealed cross-class conservation: nucleotide/amino acid similarities ranged from 71% to 99%, with the highest homology to canids (99%) and the lowest to *Cyprinus carpio* (71%) (Table 3). A UPGMA phylogenetic tree constructed using MEGA 4.0 (Figure 3) showed Arctic foxes forming a monophyletic clade with canids, while primates exhibited topological anomalies, suggesting potential lineage-specific evolution of *IGF1R* in primates. Genetic distance analysis via the Kimura 2-parameter method indicated zero net distance between Arctic foxes and canids, and a maximum distance of 0.271 with Cyprinus carpio (Table 4), aligning with vertebrate phylogenetic hierarchy.

### 3.4. Structural and Functional Analysis of Arctic Fox IGF1R Protein

#### 3.4.1. Physicochemical Properties and Hydrophobicity

**Figure 4:**
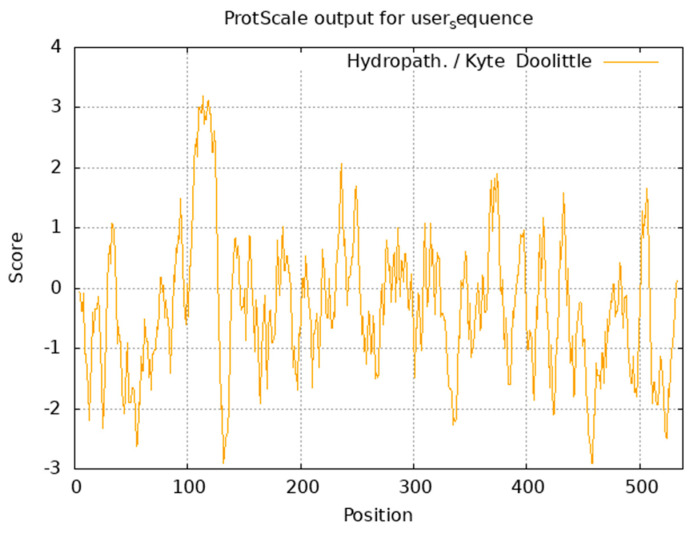
Prediction of hydrophobic structure of *IGF1R* in the Arctic fox.

ExPASy ProtParam analysis revealed the Arctic fox *IGF1R* protein (molecular formula, C2702H4203N725O805S28) has a molecular weight of 60.62 kDa, comprising 538 amino acids. Dominant residues included glutamate (Glu, 9.1%) and leucine (Leu, 9.1%), while histidine (His, 1.5%) and tryptophan (Trp, 1.5%) were least abundant. Acidic residues (Asp + Glu = 73) exceeded basic residues (Arg + Lys = 56) (Table 5), with a theoretical pI of 5.15, confirming acidic properties. The protein exhibited hydrophilicity (average GRAVY = −0.337), thermolability (instability index = 48.59), and moderate lipophilicity (aliphatic index = 81.51). Kyte–Doolittle hydrophobicity analysis identified a maximum hydrophobic peak (+3.189) at Ala114 and a minimum (−2.911) at Asp457 (Figure 4).

#### 3.4.2. Transmembrane Domain and Subcellular Localization

**Figure 5:**
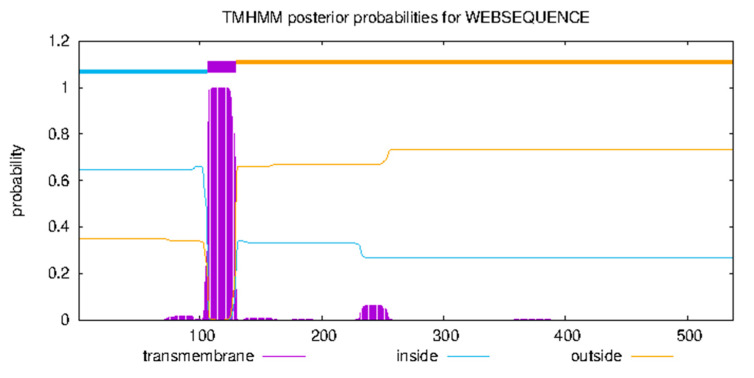
Transmembrane structure prediction of *IGF1R* protein in the Arctic fox.

Prediction indicated a single α-helix transmembrane domain (107–129 aa), with the N-terminus (1–106 aa) being cytoplasmic and the C-terminus (130–538 aa) being extracellular (Figure 5), consistent with type-II transmembrane receptors. PSORT II predicted nuclear (34.8%), cytosolic (21.7%), mitochondrial (21.7%), and secretory vesicle (8.7%) localization, suggesting dynamic involvement in transmembrane signaling and vesicle trafficking, aligning with *IGF1R*-mediated PI3K/AKT and MAPK pathway activation.

#### 3.4.3. Phosphorylation Site Prediction

**Figure 6:**
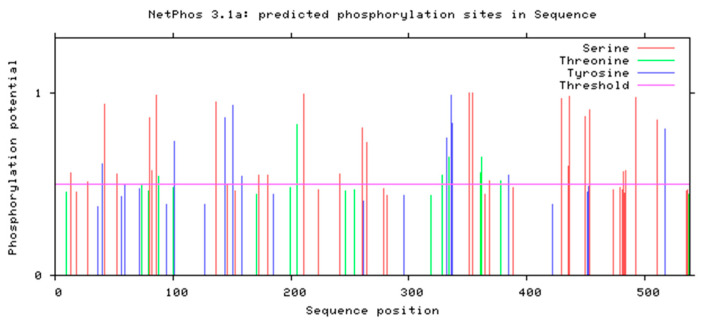
Analysis of phosphorylation potential of *IGF1R* in the Arctic fox.

NetPhos 3.1 (threshold > 0.5) predicted 27 potential phosphorylation sites: 18 serine (66.7%), 6 threonine (22.2%), and 3 tyrosine (11.1%) residues (Figure 6).

#### 3.4.4. Secondary and Tertiary Structure Prediction

**Figure 7:**
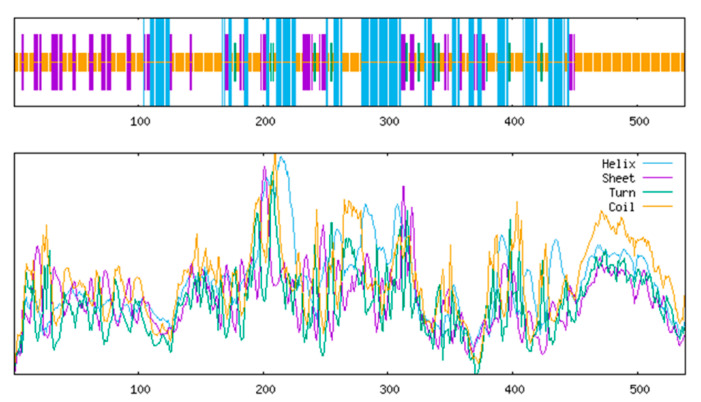
*IGF1R* secondary structure prediction. The longest vertical line is the alpha helix; the second-longest vertical line is the extended chain; the secondary short vertical line is the Beta turn; and the shortest vertical line is the random coil.

**Figure 8:**
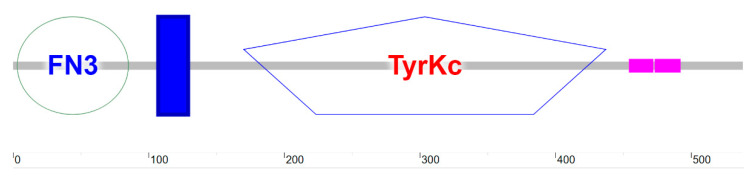
Functional domain prediction of *IGF1R* protein in the Arctic fox.

**Figure 9:**
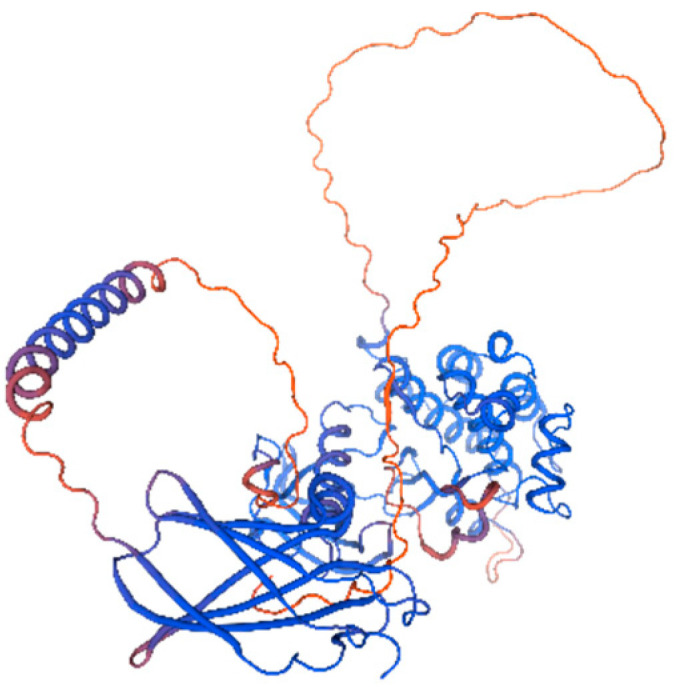
Predicted tertiary structure of Arctic fox *IGF1R* protein.

SOPMA analysis indicated dominance of random coils (52.79%) and α-helices (26.39%), with fewer extended strands (16.91%) and β-turns (3.90%) (Figure 7). SMART identified three domains: an N-terminal fibronectin type-III domain (3–85 aa) for ligand binding, a transmembrane helix (107–129 aa), and a C-terminal tyrosine kinase catalytic domain (170–437 aa) (Figure 8). SWISS-MODEL tertiary structure prediction (Figure 9) corroborated these features.

#### 3.4.5. Protein Interaction Analysis

**Figure 10:**
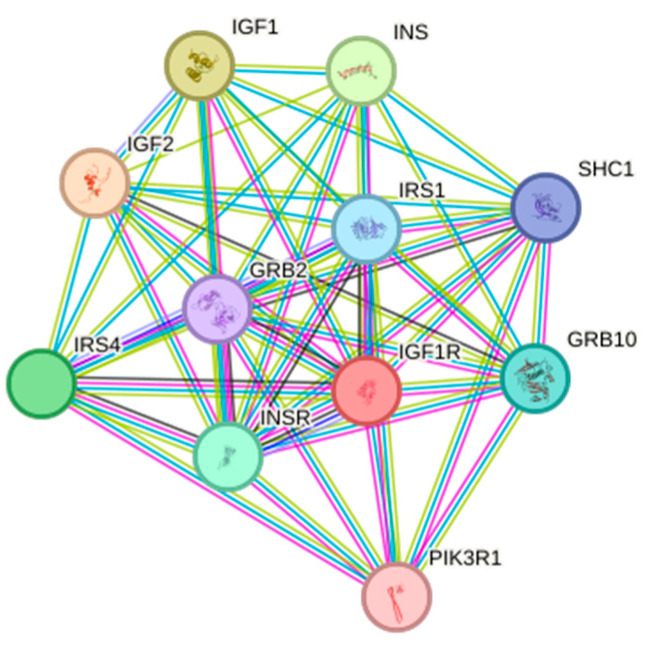
Protein interaction analysis of *IGF1R* in the Arctic fox.

STRING database analysis revealed an 11-node interaction network with high connectivity (average node degree = 9.09) and clustering (local clustering coefficient = 0.911). Core interactors included insulin signaling components (INS/IGF1/IGF2), adaptors (SHC1/GRB2/GRB10), and PI3K-AKT regulators (PIK3R1/IRS1/IRS4), enriched in cell proliferation and organ development processes (Figure 10).

### 3.5. Tissue-Specific and Spatiotemporal Expression Patterns of IGF1R Gene in the Arctic Fox

qRT-PCR showed *IGF1R* expression in 11 of 12 organs (excluding duodenum) in 6-month-old Arctic foxes, with the highest levels in the testis and the lowest in the stomach (Table 6). Temporal analysis indicated age-dependent increases in liver expression, peak testis expression at 6 months, and highest muscle expression at 4 months (Table 7).

## 4. Discussion

Insulin-like growth factor I receptor (*IGF1R*), as a core effector molecule in the growth hormone axis cascade, coordinates organismal development by maintaining cellular homeostasis and driving proliferation–differentiation programs. As a key mediator of the GH-IGFs signaling axis, it exerts pleiotropic effects in metabolic regulation [1,14,15]. Loss-of-function studies demonstrate that *IGF1R* knockout leads to embryonic muscle-developmental defects in mice [16], hepatic regeneration disorders, and pulmonary developmental abnormalities [17], while muscle-specific overexpression induces abnormal muscle hypertrophy [5]. Evolutionary analyses reveal that structural variations in and transcriptional–translational efficiency attenuation of the *IGF1R* gene constitute critical mechanisms underlying dwarfism phenotypes, validated in both murine models and human genetic cases. Compared to extensive research on pigs [8], cattle [18], sheep [19], poultry [20], and aquatic species [21], functional characterization of *IGF1R* in Arctic foxes remains largely unexplored. This study systematically elucidates its molecular features and expression regulatory patterns for the first time, providing novel perspectives on growth axis research in polar mammals.

Based on the domestic dog *IGF1R* gene (Accession No. XM_545828), primers were designed to successfully clone a partial cDNA sequence (2411 bp) of Arctic fox *IGF1R* using RT-PCR. This sequence showed high homology with mammalian *IGF1R*, with the complete CDS region (1617 bp) encoding 538 amino acids. Phylogenetic analysis revealed 99% nucleotide sequence homology between Arctic fox *IGF1R* and domestic dogs, significantly higher than homology with pigs (98%), mice (96%), and poultry (84%), consistent with mammalian taxonomic relationships. Notably, *IGF1R* evolutionary trees exhibited deviations from traditional classifications in primates, suggesting potential functional divergence of the IGF family in primates. Phosphorylation prediction identified 27 potential phosphorylation sites, with structural domain remodeling induced by site-specific phosphorylation potentially regulating multi-pathway signal transduction networks [22]. Six Ser sites localized in the tyrosine kinase catalytic domain may mediate intracellular signaling through autophosphorylation [23]. Functional domain analysis demonstrated that Arctic fox *IGF1R* contains an 83-amino-acid fibronectin type-III (FN3) domain mediating DNA/heparin binding [24], while its transmembrane region (107–129 aa) confirms its identity as a transmembrane receptor. The tyrosine kinase (*TyrKc*) domain harbors multiple binding sites, enabling diverse catalytic reactions [25]. Protein interaction network analysis revealed strong interactions between *IGF1R* and 11 proteins, including *IGF1*/*IGF2*/*INS*. *IGF1*, as a key member of the insulin-like growth factor family, plays vital roles in growth regulation, skeletal muscle development, cellular proliferation/differentiation, tissue repair, and metabolic modulation. Importantly, *IGF1R* not only mediates IGF ligand signaling but also critically regulates embryonic development, cell growth/differentiation, mitosis, embryonic survival, and metabolic adaptation [26]. *IGF1* drives evolutionary processes by modulating growth rates, body size differentiation, and physiological adaptability. *IGF1R*-*IGF2* binding triggers tyrosine kinase domain autophosphorylation, activating PI3K/AKT and MAPK/ERK signaling cascades to promote metabolism, proliferation, and differentiation [26]. Genomic analyses identify numerous SNPs in porcine and ovine *IGF1R* genes, with specific variants significantly influencing growth traits through receptor conformation and expression levels, contributing to breed-specific size variations [27,28]. Notably, bovine *IGF1R* polymorphisms correlate strongly with milk-production traits (milk fat/protein content), highlighting its potential as a molecular marker in dairy cattle breeding [29]. These findings collectively underscore the central role of IGF signaling in animal growth regulation.

Using qRT-PCR (with *GAPDH* as reference), tissue-specific expression profiling of *IGF1R* in 6-month-old Arctic foxes revealed differential expression across the 12 organ tissues, except duodenum. Testicular *IGF1R* mRNA levels significantly exceeded other organ tissues, followed by kidney, muscle, liver, pituitary, brain, spleen, lung, heart, pancreas, and stomach. Notably, the absence of *IGF1R* expression in duodenal tissue may indicate its limited physiological role in this organ [30]; suggest predominant reliance on alternative signaling pathways for growth regulation [31]; or reflect technical limitations, such as undetectable expression levels (below qPCR detection threshold) or RNA degradation during preservation, affecting assay sensitivity.

Developmental expression analysis in liver, testis, and muscle tissues at 2-, 4-, and 6-month-old stages demonstrated age-dependent expression patterns. Hepatic expression showed progressive upregulation (2-month-old levels significantly lower than subsequent stages), aligning with developmental patterns observed in Nanjiang yellow goats [27]. Testicular expression peaked at 6 months (6-month-old > 2-month-old > 4-month-old), while muscular expression reached maximum at 4 months (4-month-old > 6-month-old > 2-month-old). The inclusion of testicular tissue in developmental studies stemmed from its highest expression among 12 organ tissues at 6 months, suggesting *IGF1R* involvement in Arctic fox reproductive regulation—a hypothesis reinforced by sustained high expression across 2- and 4-month-old testicular samples.

The spatiotemporal expression of *IGF1R* across tissues carries significant biological implications: In liver tissue, *IGF1R* expression increased with age, reflecting its core role in energy metabolism and growth substrate synthesis. As the primary site of *IGF-1* synthesis, upregulated receptor expression in liver may enhance responsiveness to circulating *IGF-1*, promoting glycogen synthesis and protein accumulation to support rapid growth in juvenile foxes [32]. Testicular tissue expression peaked at 6 months old, suggesting *IGF1R*’s potential involvement in Arctic fox sexual maturation by regulating spermatogonial proliferation and testosterone synthesis, thereby influencing reproductive organ development and breeding performance [33]. The muscular expression peak at 4 months old coincided with the rapid growth phase of skeletal muscle, demonstrating that *IGF1R* directly regulates muscle growth efficiency through satellite cell differentiation and myofiber hypertrophy—consistent with its established role in mammalian myogenic differentiation [17].

As a transmembrane tyrosine kinase receptor, *IGF1R* ubiquitously localizes on cell membranes, mediating proliferation, differentiation, and anti-apoptotic functions through *IGF1*/*IGF2* binding [34]. Current research predominantly focuses on its roles in tumorigenesis and therapeutic resistance, whereas tissue-specific functions in Arctic foxes require further exploration via proteomic interactome mapping and conditional knockout models. This study provides foundational insights into Arctic fox *IGF1R* expression profiles and structural characteristics, facilitating future investigations into molecular mechanisms underlying growth trait regulation.

## 5. Conclusions

This study successfully cloned the full CDS (1617 bp) of *IGF1R* in the Arctic fox, with phylogenetic analysis confirming its high conservation (99% homology) within canines. The receptor exhibited characteristic tyrosine kinase features, including a fibronectin type-III ligand-binding domain, transmembrane region, and catalytic domain, with acidic hydrophilic properties (pI = 5.15). Spatiotemporal expression analysis revealed tissue-specific and developmental stage-specific regulation: *IGF1R* expression was highest in testicular tissue, while dynamic patterns were observed across ages—liver expression increased progressively with postnatal development, aligning with enhanced metabolic demands; testicular expression peaked in 6-month-old individuals, suggesting roles in sexual maturation; and muscular expression maxima at 4 months of age corresponded to rapid skeletal muscle growth.

## Figures and Tables

**Table 1:** Primer information.

Primers	Sequences	Annealing Temperature	Application
*IGF1R-1*	F: GCAGAAGGAGCAGATGACATTC R: GAGGTGCGAGGACTGGATTAT	54	PCR amplification
*IGF1R-2*	F: AATCCAGTCCTCGCACCTC R: AGGAGGCTGTGGCTAGATTG	60	PCR amplification
*IGF1R-3*	F: GGAGTTCTAACAATCTAGCC R: ACCTGCGTGAATGTAAGAC	40	PCR amplification
*IGF1R*	F: GTCCTTCGCTTCGTTATG R: CTTGTTCTCCTCGCTGTAG	60	qRT-PCR
*GAPDH*	F: GGGTGATGCTGGTGCTG R: ACGGTCTTCTGGGTGGC	60	qRT-PCR

**Table 2:** Bioinformatics analysis software and function.

Analysis Tools	Websites	Function Prediction
ExPASy	https://web.expasy.org/protscale/ (accessed on 24 February 2025)	Physicochemical properties
ProtSca	https://web.expasy.org/protscale (accessed on 24 February 2025)	Hydrophilicity/hydrophobicity
Novopro	https://www.novopro.cn/tools/tmhmm.html (accessed on 24 February 2025)	Transmembrane domain
SMART	http://smart.embl-heidelberg.de/ (accessed on 24 February 2025)	Functional domain
NetPhos-3.1	https://services.healthtech.dtu.dk/services/NetPhos-3.1/ (accessed on 25 February 2025)	Phosphorylation sites
SOPMA	https://npsa.lyon.inserm.fr/cgi-bin/ (accessed on 25 February 2025)	Secondary structure
SWISS-MODEL	https://swissmodel.expasy.org/ (accessed on 25 February 2025)	Tertiary structure
STRING	https://cn.string-db.org/ (accessed on 25 February 2025)	Protein–protein interaction

**Table 3:** Amino acid homology between Arctic fox *IGF1R* and *IGF1R*s of other species.

Species	Similarity of Mature	Amino Acid Number	GenBank Accession No.
*Canis familiaris*	99%	538	XP_545828
*Sus scrofa*	98%	538	NP_999337
*Equus caballus*	98%	538	XP_001489815
*Bos Taurus*	97%	538	XP_606794
*Homo sapiens*	97%	538	EAX02222
*Macaca mulatta*	97%	538	XP_001100407
*Rattus norvegicus*	93%	538	AAA41392
*Mus musculus*	96%	538	NP_034643
*Gallus gallus*	84%	538	NP_990363
*Coturnix japonica*	84%	538	BAF73401
*Xenopus laevis*	79%	538	CAA90517
*Cyprinus carpio*	71%	538	AAN52152

**Table 4:** Interspecific genetic distances of the IGF1R gene calculated using the Kimura 2-parameter method.

	Quail	Chick	Monkey	Human	Pig	Cattle	Horse	Fox	Dog	Rat	Mouse	Xenopus
Chick	0.006											
Monkey	0.134	0.132										
Human	0.137	0.134	0.002									
Pig	0.128	0.125	0.008	0.010								
Cattle	0.134	0.132	0.018	0.020	0.014							
Horse	0.134	0.132	0.012	0.014	0.008	0.016						
Fox	0.137	0.134	0.018	0.020	0.014	0.024	0.010					
Dog	0.137	0.134	0.018	0.020	0.014	0.024	0.010	0.000				
Rat	0.146	0.144	0.032	0.035	0.028	0.035	0.028	0.032	0.032			
Mouse	0.148	0.146	0.037	0.039	0.032	0.039	0.032	0.037	0.037	0.004		
Xenopus	0.165	0.157	0.203	0.205	0.198	0.205	0.203	0.205	0.205	0.208	0.210	
Carp	0.248	0.240	0.276	0.274	0.271	0.271	0.274	0.271	0.271	0.287	0.284	0.266

**Table 5:** Physicochemical properties of *IGF1R* protein in Alopex.

Amino Acids	Number	Frequency (%)	Amino Acids	Number	Frequency (%)
Ala (A)	31	5.8	Lys (K)	23	4.3
Arg (R)	33	6.1	Met (M)	22	4.1
Asn (N)	27	5.0	Phe (F)	20	3.7
Asp (D)	24	4.5	Pro (P)	39	7.2
Cys (C)	6	1.1	Ser (S)	40	7.4
Gln (Q)	14	2.6	Thr (T)	17	3.2
Glu (E)	49	9.1	Trp (W)	8	1.5
Gly (G)	40	7.4	Tyr (Y)	22	4.1
His (H)	8	1.5	Val (V)	41	7.6
Ile (I)	25	4.6	Pyl (O)	0	0
Leu (L)	49	9.1	Sec (U)	0	0

**Table 6:** Expression characteristics of Arctic fox *IGF1R* gene in 11 organ tissues.

Histophysiology	GAPDH (Ct1)	*IGF1R* (Ct2)	Relative Expression (2^Ct1-Ct2^)	Brain Expression-Normalized (Set to 1)
Liver	22.123	23.060	0.522 ± 0.109	1.906 ± 0.398
Spleen	23.057	24.960	0.267 ± 0.085	0.976 ± 0.309
Kidney	22.873	23.520	0.639 ± 0.230	2.331 ± 0.840
Lung	19.745	22.150	0.189 ± 0.078	0.690 ± 0.286
Stomach	20.323	26.160	0.018 ± 0.003	0.064 ± 0.011
Pancreas	18.157	21.137	0.127 ± 0.107	0.463 ± 0.389
Brain	19.767	21.633	0.274 ± 0.155	1.000 ± 0.565
Pituitary gland	20.793	21.853	0.480 ± 0.154	1.750 ± 0.561
Testis	22.333	20.883	2.732 ± 0.486	9.971 ± 1.773
Heart	21.737	24.197	0.182 ± 0.052	0.663 ± 0.190
Muscle	20.443	21.273	0.563 ± 0.057	2.053 ± 0.208

**Table 7:** Spatiotemporal expression characteristics of the *IGF1R* gene in Arctic fox organ tissues.

Histophysiology	GAPDH (Ct1)	*IGF1R* (Ct2)	Relative Expression (2^Ct1-Ct2^)	Muscle Expression in 4-Month-Old Male Foxes-Normalized (Set to 1)
2-month-old male fox liver	22.030	26.527	0.044 ± 0.017	0.043 ± 0.016
4-month-old male fox liver	18.353	19.450	0.467 ± 0.095	0.451 ± 0.090
6-month-old male fox liver	22.123	23.060	0.522 ± 0.127	0.504 ± 0.120
2-month-old male fox testis	21.120	19.760	2.567 ± 0.264	2.480 ± 0.250
4-month-old male fox testis	21.107	20.020	2.124 ± 0.323	2.052 ± 0.306
6-month-old male fox testis	22.333	20.883	2.732 ± 0.225	2.640 ± 0.213
2-month-old male fox muscle	20.220	22.993	0.146 ± 0.008	0.141 ± 0.007
4-month-old male fox muscle	18.460	18.410	1.035 ± 0.264	1.000 ± 0.250
6-month-old male fox muscle	20.443	21.273	0.563 ± 0.066	0.544 ± 0.063

## Data Availability

Should you require any further data, please do not hesitate to contact us. For the sake of data privacy, this information is not available to the public.

## Abbreviations

Key Symbol Comparison Table

IGFIR	Insulin-like growth factor 1 receptor
cDNA	Complementary DNA
IGF1	Insulin like growth factor 1
IGF2	Insulin like growth factor 2
MC1R	Melanocortin 1 receptor
GAPDH	Glyceraldehyde-3-phosphate dehydrogenase
CDS	Coding sequences
ORF	Open reading frame
DEPC	Diethylpyrocarbonate
UTR	Untranslated regions
PI	Isoelectric point
LSM	Least squares method
SNP	Single-nucleotide polymorphism
FSH	Follicle-stimulating hormone
GH	Growth hormone
